# Sex differences in functional limitations and the role of socioeconomic factors: a multi-cohort analysis

**DOI:** 10.1016/S2666-7568(21)00249-X

**Published:** 2021-12

**Authors:** Mikaela Bloomberg, Aline Dugravot, Benjamin Landré, Annie Britton, Andrew Steptoe, Archana Singh-Manoux, Séverine Sabia

**Affiliations:** a Department of Epidemiology and Public Health, UCL Institute of Epidemiology and Health Care, University College London, London, UK; b Department of Behavioural Science and Health, University College London, London, UK; c Université de Paris, Inserm U1153, Epidemiology of Ageing and Neurodegenerative Diseases, Paris, France

## Abstract

**Background:**

Women are more likely to have functional limitations than are men, partly because of greater socioeconomic disadvantage. However, how sex differences vary by severity of functional limitations remains unclear. We examined sex differences in functional limitations, with attention to socioeconomic factors and severity of limitations.

**Methods:**

Longitudinal data on limitations in basic activities of daily living (ADL) and instrumental activities of daily living (IADL) and mobility activities were drawn from 62 375 participants from 14 countries. For ADL, IADL, and mobility, participants were categorised based on number of limited activities (0, 1, 2, or ≥3). Sex differences in limitations in four birth cohorts (1895–1929, 1930–38, 1939–45, and 1946–60) were analysed before and after adjustment for socioeconomic factors (education and labour force status).

**Findings:**

The prevalence of IADL and ADL limitations was higher in women than in men. After adjustment for socioeconomic factors, this sex difference was attenuated. The sex difference in IADL limitations at age 75 years (in the 1895–1929 cohort) was 3·7% before adjustment for socioeconomic factors (95% CI 2·6–4·7) and 1·7% (1·1–2·2) after adjustment. For ADL, the sex difference in limitations at age 75 years (in the 1895–1929 cohort) was 3·2% (2·3–4·1) before adjustment for socioeconomic factors and 1·4% (0·9–1·8) after adjustment. Sex differences in mobility limitations (16·1%, 95% CI 14·4–17·7) remained after adjustment for socioeconomic factors (14·3%, 12·7–15·9). After age 85 years, women were more likely to have three or more IADL or mobility limitations and men were more likely to have one or two limitations.

**Interpretation:**

Socioeconomic factors largely explain sex differences in IADL and ADL limitations but not mobility. Sex differences in mobility limitations in midlife are important targets for future research and interventions.

**Funding:**

National Institute on Aging, UK National Institute for Health Research, European Commission, and US Social Security Administration.

## Introduction

Disability at older ages is associated with institutionalisation, higher health-care costs, increased mortality risk, and poorer quality of life.^1^ The prevalence of disability is higher in women, including functional limitations in basic activities of daily living (ADL), instrumental activities of daily living (IADL),^2^ and mobility limitations.^3^ Sex differences in functional limitations are hypothesised to result from a combination of women surviving longer with limitations and other biological and socioeconomic differences between men and women.^4^, ^5^ Although mobility limitations tend to precede IADL and ADL limitations,^6^ more information on sex differences in the timing and nature of the disablement process would be valuable to plan targeted prevention policies to preserve independent living and quality of life for older adults.^1^

During the 20th century women have had progressively more access to education and have become more likely to enter the labour force.^7^ Given this improvement, it is important to consider the historical context in analyses of sex differences in disability.^2^ There is evidence of a reduction in the prevalence of functional limitations with increasing birth year,^8^, ^9^, ^10^, ^11^, ^12^, ^13^, ^14^, ^15^ particularly for women.^10^, ^11^, ^12^, ^16^ Although socioeconomic factors are likely to underlie variations in sex differences in disability across birth cohorts, the role of such factors has not been well explored in this context. There are other limitations in this body of knowledge. First, studies of sex differences in functional limitations by birth cohort are based on small samples^8^, ^10^, ^11^, ^16^, ^17^ and cover a restricted range of birth years.^8^, ^10^, ^11^, ^14^, ^16^, ^18^ Second, limitations are often examined dichotomously^10^, ^13^, ^19^ or combined into indices^14^ that do not necessarily translate into easily interpretable measures of disability. Indices tend to combine several measures of disability so that a unit increase in a disability index is difficult to interpret. Dichotomous categorisation of functional limitations includes individuals with one or several limitations in the same category, thus failing to consider potential variation in sex differences by severity of limitations.

To gain further understanding of sex differences in disability, we used longitudinal data pooled from four cohort studies of individuals from 14 countries aged 50–107 years to investigate the role of socioeconomic factors in sex differences in mobility, IADL, and ADL limitations across birth cohorts from 1895–1960, and to examine how sex differences vary by severity of limitations for each of the three measures (mobility, IADL, and ADL).

**Research in context**

**Evidence before this study**

We searched PubMed for articles published from inception up to June 6, 2021, using the search terms “activities of daily living”, “mobility limitation”, “functional status”, “sex”, “disability”, “birth cohort”, “socioeconomic factors”, and “aging”, with no language restrictions. Previous studies have suggested that disability assessed using functional limitations is more prevalent among women than men and socioeconomic factors are thought to play a role. Differences between men and women in socioeconomic circumstances have decreased considerably over the 20th century; accordingly, there is evidence that sex differences in functional limitations have decreased in consecutive birth cohorts. Although some studies have examined the role of socioeconomic factors in sex differences, including consideration of birth cohort, these studies were either undertaken in small samples or based on dichotomous categorisations of functional limitations, which do not consider the severity of limitations based on the number of limitations.

**Added value of this study**

Developing targeted prevention policies to preserve independent living and quality of life for older adults requires an understanding of sex differences in the timing and nature of functional limitations. Sex differences in functional limitations are frequently examined using prevalence of one or more basic activities of daily living (ADL) or instrumental activities of daily living (IADL) or mobility limitations. Our findings extend current evidence by showing that at older ages, when limitations are frequent, prevalence of limitations was not only higher among women but women were also more likely to have a greater number of limitations than were men. After accounting for socioeconomic factors, we found sex differences to be considerably attenuated for limitations in ADL and IADL, which typically occur in later life, and sex differences in ADL were no longer evident in recent birth cohorts. However, across all birth cohorts and ages, women reported having more mobility limitations, starting in midlife.

**Implications of all the available evidence**

Increasing socioeconomic equity between men and women is implicated in reducing sex differences in disability occurring at older ages. Nonetheless, identifying and targeting drivers of sex disparities in mobility limitations from middle age onward remains an important focus for reduction of sex disparities in disability, as mobility limitations are thought to be the first step in progressive decline in functional independence.

## Methods

### Data sources

Data were taken from the English Longitudinal Study of Ageing (ELSA),^20^ the Irish Longitudinal Study on Ageing (TILDA),^21^ the Survey of Health, Ageing and Retirement in Europe (SHARE),^22^ and the Health and Retirement Study (HRS)^23^ in populations from England, Ireland, 11 European countries (appendix p 11), and the USA, respectively. These studies have been designed to facilitate cross-national comparisons. People living in institutions were included in the sampling frame for SHARE, but not for ELSA, HRS, or TILDA. In all studies, participants who were institutionalised during follow-up were included in follow-up waves. Further details of survey design are discussed at length elsewhere.^20^, ^21^, ^22^, ^23^ All studies obtained ethical approval from relevant local research ethics committees.

The present study included waves 1–9 of ELSA (surveyed every 2 years from 2002–03 to 2018–19), waves 1, 3, and 4 of TILDA (2009–11, 2014–15, and 2016, respectively), waves 1, 2, and 4–7 of SHARE (2004–05, 2006–07, 2010–11, 2013, 2015, and 2017, respectively), and waves 5–13 of HRS (2000, and for 2-year periods 2002–03 to 2016–17), so that years of follow-up were comparable between studies (appendix p 12). Data from participants in these studies who were older than 50 years at the baseline wave of the present study were pooled for analysis. As participants in ELSA and TILDA who were older than 80 years or 90 years, respectively, at wave 1 had their ages coded as 80 years or 90 years without further precision, these participants were excluded from the present analyses.

### Sex and covariates

Sex was self-reported as male or female. Birth cohorts included pre-Depression era (1895-1929), Depression era (1930–38), World War 2 (1939–45) and post-War (1946–60) cohorts.

Other sociodemographic covariates included age, region (western Europe, northern Europe, southern Europe, or North America), study (SHARE, ELSA, TILDA, or HRS [equivalent to North America region category]), and marital status (married or partnered *vs* not married or partnered).

Socioeconomic factors included education, with categories derived from the International Standard Classification of Education 1997 (less than upper secondary or some high school, upper secondary or high school diploma and vocational training, or university degree and above) and labour force status (employed or self-employed, retired, unemployed or unable to work, or homemaker).

### Functional limitations

Three measures of functional limitation were examined: ADL, IADL, and mobility limitations. Each functional measure was composed of six activities (appendix p 13). Participants were considered limited for a given activity if they answered “yes” when asked whether they had experienced difficulty performing the activity for longer than 3 months because of a physical, mental, emotional, or memory problem.

For each functional measure, the number of limited activities was summed to yield a score from 0 to 6. Participants with a score of 1 or greater were considered limited for the given functional measure. Severity of limitations for each functional measure was examined using zero, one, two, or three or more limited activities.

### Statistical analysis

Participant characteristics were described in the pooled sample and by birth cohort, with differences between men and women assessed using Pearson's χ^2^ test and *t* test for categorical and continuous variables, respectively. We plotted the observed proportion of participants with at least one mobility, IADL, and ADL limitation by sex and age group in each study separately.

We used mixed effects ordinal logistic models to examine sex differences in functional limitation severity, including random intercept and slope, with an unstructured covariance matrix to account for intraindividual clustering. We used age as the time scale. For each of the three functional limitation measures, the initial model included sex, age, birth cohort, sex by age, birth cohort by age, region, study, and time-varying marital status. Higher order interactions with age were then examined for each of these covariates and retained in the model if shown to be significant based on the Wald test (α=0·050). The model was then adjusted for education and higher order interactions of education with age if significant. Time-varying labour force status was further added to the model. In supplementary analyses, we did the following: adding self-reported chronic conditions (high blood pressure, diabetes, cancer, lung disease, psychiatric illness, arthritis, and cardiovascular disease [including heart attack and stroke]) as time-varying covariates; and including sex by region and sex by region by age interaction terms, and then stratified by region. To facilitate interpretation of results, the sex difference (female–male) in the probability of having zero, one, two, or three or more limitations in mobility, IADL, and ADL was derived from the ordinal models, estimated every 5 years from age 50 to 100 years. The probability of having at least one limitation was also calculated as 1 minus the probability of having zero limitations.

All statistical analyses were done in Stata version 16.1 or 17.0.

### Role of the funding source

The funders of the study had no role in study design, data collection, data analysis, data interpretation, or writing of the report.

## Results

Of 67 448 participants at the baseline waves of ELSA (11 391 at wave 1 in 2002; appendix p 3), TILDA (8504 at wave 1 in 2010; appendix p 4), SHARE (27 975 at wave 1 in 2004; appendix p 5), and HRS (19 578 at wave 5 in 2000; appendix p 6), 2036 (3·0%) participants were younger than 50 years and 721 (1·1%) participants had their age set at 90 years (ELSA) or 80 years (TILDA) at baseline, leading us to exclude them from the analysis. Of the remaining 64 691 participants, 1404 (2·2%) were missing either ADL, IADL, or mobility data for all waves, and 912 (1·4%) were missing data on covariates for all waves, resulting in an analytic sample of 62 375 participants. 375 (0·6%) of 62 375 participants were resident in institutions at baseline in the present study. Follow-up was from January, 2000, to January, 2019, with a median follow-up of 7 years (IQR 2–13).

At baseline, women were older than men (mean age 65·2 years, SD 10·2 *vs* 64·8 years, 9·5 in men; p<0·0001) and were less likely to have education above secondary level, to be married, and to be employed than men (p<0·0001 for all comparisons; table 1). The proportion of participants with education above secondary level increased in later birth cohorts (p_trend_<0·0001), particularly for women (from 678 [8·2%] of 8257 participants in the 1895–1929 birth cohort to 1916 [19·7%] of 9740 participants in the 1946–60 birth cohort; in men the corresponding numbers were 978 [16·8%] of 5818 participants in the 1895–1929 birth cohort and 1876 [23·9%] of 7852 participants in the 1946–60 birth cohort; table 1). Among women who were not retired (employed or self-employed, unemployed or unable to work, and homemakers), those born in more recent birth cohorts were more likely to be employed than were those in earlier birth cohorts (p_trend_<0·0001).

**Table 1:** Baseline population characteristics for men and women

		**Overall**		**1895–1929 birth cohort**		**1930–38 birth cohort**		**1939–45 birth cohort**		**1946–60 birth cohort**	
		Men (n=27 923)	Women (n=34 452)	Men (n=5818)	Women (n=8257)	Men (n=7602)	Women (n=8648)	Men (n=6651)	Women (n=7807)	Men (n=7852)	Women (n=9740)
Age at baseline, years		64·8 (9·5)	65·2 (10·2)	78·5 (5·1)	79·3 (5·4)	68·1 (3·8)	68·0 (3·8)	61·1 (3·6)	60·8 (3·6)	54·6 (3·2)	54·4 (3·3)
Cohort											
	ELSA	4957 (17·8%)	5682 (16·5%)	1190 (20·5%)	1557 (18·9%)	1362 (17·9%)	1435 (16·6%)	1164 (17·5%)	1258 (16·1%)	1241 (15·8%)	1432 (14·7%)
	TILDA	3461 (12·4%)	4052 (11·8%)	..	..	587 (7·7%)	660 (7·6%)	806 (12·1%)	819 (10·5%)	2068 (26·3%)	2573 (26·4%)
	SHARE	12 140 (43·5%)	14 367 (41·7%)	2082 (35·8%)	2904 (35·2%)	3055 (40·2%)	3363 (38·9%)	2909 (43·7%)	3293 (42·2%)	4094 (52·1%)	4807 (49·4%)
	HRS	7365 (26·4%)	10 351 (30·0%)	2546 (43·8%)	3796 (46·0%)	2598 (34·2%)	3190 (36·9%)	1772 (26·6%)	2437 (31·2%)	449 (5·7%)	928 (9·5%)
Region											
	Northern Europe	2130 (7·6%)	2412 (7·0%)	405 (7·0%)	490 (5·9%)	501 (6·6%)	527 (6·1%)	527 (7·9%)	600 (7·7%)	697 (8·9%)	795 (8·2%)
	Western Europe	15 146 (54·2%)	17 643 (51·2%)	2299 (39·5%)	3130 (37·9%)	3595 (47·3%)	3923 (45·4%)	3564 (53·6%)	3850 (49·3%)	5688 (72·4%)	6740 (69·2%)
	Southern Europe	3282 (11·8%)	4046 (11·7%)	568 (9·8%)	841 (10·2%)	908 (11·9%)	1008 (11·7%)	788 (11·8%)	920 (11·8%)	1018 (13·0%)	1277 (13·1%)
	North America	7365 (26·4%)	10 351 (30·0%)	2546 (43·8%)	3796 (46·0%)	2598 (34·2%)	3190 (36·9%)	1772 (26·6%)	2437 (31·2%)	449 (5·7%)	928 (9·5%)
Marital status											
	Not married or partnered	5340 (19·1%)	12 983 (37·7%)	1590 (27·3%)	5337 (64·6%)	1318 (17·3%)	3372 (39·0%)	1094 (16·4%)	2136 (27·4%)	1338 (17·0%)	2138 (22·0%)
	Married or partnered	22 583 (80·9%)	21 469 (62·3%)	4228 (72·7%)	2920 (35·4%)	6284 (82·7%)	5276 (61·0%)	5557 (83·6%)	5671 (72·6%)	6514 (83·0%)	7602 (78·0%)
Education											
	Below secondary	10 507 (37·6%)	15 240 (44·2%)	2784 (47·9%)	4675 (56·6%)	3156 (41·5%)	4272 (49·4%)	2345 (35·3%)	3159 (40·5%)	2222 (28·3%)	3134 (32·2%)
	Secondary	11 719 (42·0%)	14 527 (42·2%)	2056 (35·3%)	2904 (35·2%)	3081 (40·5%)	3461 (40·0%)	2828 (42·5%)	3472 (44·5%)	3754 (47·8%)	4690 (48·2%)
	Above secondary	5697 (20·4%)	4685 (13·6%)	978 (16·8%)	678 (8·2%)	1365 (18·0%)	915 (10·6%)	1478 (22·2%)	1176 (15·1%)	1876 (23·9%)	1916 (19·7%)
Labour force status											
	Employed or self-employed	9800 (35·1%)	8890 (25·8%)	176 (3·0%)	139 (1·7%)	1017 (13·4%)	788 (9·1%)	2835 (42·6%)	2513 (32·2%)	5772 (73·5%)	5450 (56·0%)
	Unemployed or unable to work	1804 (6·5%)	1976 (5·7%)	62 (1·1%)	243 (2·9%)	204 (2·7%)	233 (2·7%)	664 (10·0%)	538 (6·9%)	874 (11·1%)	962 (9·9%)
	Retired or semi-retired	16 159 (57·9%)	15 165 (44·0%)	5554 (95·5%)	5707 (69·1%)	6344 (83·5%)	5590 (64·6%)	3114 (46·8%)	2854 (36·6%)	1147 (14·6%)	1014 (10·4%)
	Homemaker	160 (0·6%)	8421 (24·4%)	26 (0·4%)	2168 (26·3%)	37 (0·5%)	2037 (23·6%)	38 (0·6%)	1902 (24·4%)	59 (0·8%)	2314 (23·8%)

Data are mean (SD) or n (%). ELSA=English Longitudinal Study of Ageing. TILDA=The Irish Longitudinal Study on Ageing. SHARE=Survey of Health, Ageing and Retirement in Europe. HRS=Health and Retirement Study.

**Figure 1:**
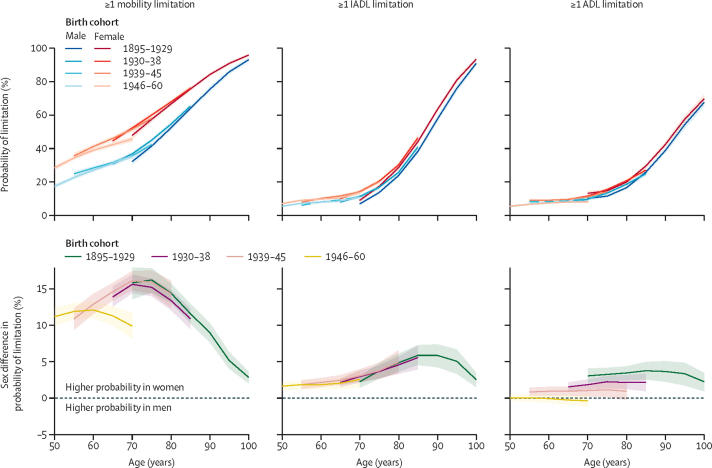
Sex differences in the probability of ≥1 mobility, IADL, and ADL limitation The top panels show the probability of having ≥1 functional limitation plotted by age for men and women in each birth cohort. The bottom panels show the sex difference in the probability of having ≥1 functional limitation. A positive value indicates women have a greater probability than men. Predicted probabilities are based on models adjusted for sex, age, birth cohort, and their interactions, marital status, study, and region and plotted for reference categories for all covariates. ADL=activities of daily living. IADL=instrumental activities of daily living.

Functional limitations increased with age for all three measures for both sexes (figure 1). Mobility limitations increased consistently from age 50 years, and IADL and ADL limitations increased slowly between ages 50 years and 70 years and then rapidly thereafter. Plots of observed data in each cohort study separately yielded broadly similar results (appendix p 7) to those in the pooled analyses.

The probability of mobility limitations increased from 17·1% (95% CI 16·1–18·2) at 50 years to 93·1% (91·7–94·4) at 100 years in men, and from 28·2% (27·0–29·5) to 95·8% (95·0–96·7) in women. Between the ages of 50 years and 100 years, IADL limitations increased from 5·1% (4·6–5·7) to 90·8% (89·4–91·2) in men and from 6·7% (6·1–7·3) to 93·2% (92·2–94·4) in women, and ADL limitations increased from 5·1% (4·6–5·7) to 67·5% (64·9–70·0) in men and from 5·2% (4·6–5·7) to 69·6% (67·2–72·0) in women.

Women were more likely to have IADL and mobility limitations, irrespective of birth cohort and age (figure 1). Sex differences in mobility limitations increased until age 70 years and until age 90 years in IADL limitations and decreased thereafter. Sex differences in ADL limitations remained substantially similar with age but varied by birth cohort (p<0·0001 for the sex by birth cohort interaction). The sex difference in ADL limitations, for which women were more likely to be limited, decreased in recent birth cohorts: at age 75, the sex difference in ADL limitations was 3·2% (95% CI 2·3–4·1) in the 1895–1929 birth cohort, 2·2% (1·3–3·0) in the 1930–38 birth cohort, and 1·1% (0·1–2·1) in the 1939–45 birth cohort (table 2). No sex differences were observed in the 1946–60 birth cohort between ages 50 years and 70 years (the ages for which data were available). This decrease in sex differences reflects a larger decrease in ADL limitations among women than in men in more recent birth cohorts.

**Table 2:** Role of socioeconomic factors in sex differences in mobility, IADL, and ADL limitations by birth cohort

	**At age 65 years**			**At age 75 years**			**At age 85 years**		
	Minimally adjusted*	Additionally adjusted for education	Additionally adjusted for labour force status	Minimally adjusted*	Additionally adjusted for education	Additionally adjusted for labour force status	Minimally adjusted*	Additionally adjusted for education	Additionally adjusted for labour force status
**≥1 mobility limitation**									
1895–1929	..	..	..	16·1 (14·4 to 17·7)	14·6 (13·0 to 16·3)	14·3 (12·7 to 15·9)	11·5 (10·2 to 12·8)	11·1 (9·8 to 12·5)	11·9 (10·4 to 13·3)
1930–38	13·8 (12·5 to 15·2)	12·1 (10·8 to 13·4)	11·3 (10·1 to 12·5)	15·1 (13·9 to 16·3)	14·0 (12·7 to 15·2)	13·6 (12·4 to 14·9)	10·8 (9·4 to 12·2)	10·4 (9·0 to 11·9)	11·0 (9·5 to 12·6)
1939–45	14·5 (13·3 to 15·7)	13·1 (12·0 to 14·3)	12·2 (11·1 to 13·3)	15·8 (14·2 to 17·4)	14·8 (13·2 to 16·4)	13·9 (12·4 to 15·5)	..	..	..
1946–60	11·2 (9·9 to 12·5)	10·5 (9·2 to 11·7)	9·3 (8·1 to 10·5)	..	..	..	..	..	..
p value for sex difference by birth cohort	0·0006	0·010	0·0014	0·63	0·68	0·81	0·47	0·48	0·43
**≥1 IADL limitation**									
1895–1929	..	..	..	3·7 (2·6 to 4·7)	2·6 (1·6 to 3·5)	1·7 (1·1 to 2·2)	5·8 (4·5 to 7·2)	4·8 (3·5 to 6·1)	4·3 (3·2 to 5·5)
1930–38	2·1 (1·5 to 2·6)	1·4 (0·9 to 1·8)	0·8 (0·5 to 1·1)	3·6 (2·7 to 4·4)	2·6 (1·8 to 3·4)	1·7 (1·2 to 2·3)	5·5 (3·9 to 7·1)	4·5 (3·0 to 6·0)	3·8 (2·4 to 5·1)
1939–45	2·4 (1·8 to 3·1)	1·8 (1·2 to 2·4)	1·0 (0·7 to 1·4)	3·7 (2·6 to 4·8)	2·9 (1·9 to 4·0)	1·8 (1·1 to 2·5)	..	..	..
1946–60	2·0 (1·2 to 2·7)	1·5 (0·8 to 2·2)	0·8 (0·4 to 1·3)	..	..	..	..	..	..
p value for sex difference by birth cohort	0·57	0·53	0·58	0·97	0·87	0·95	0·71	0·77	0·48
**≥1 ADL limitation**									
1895–1929	..	..	..	3·2 (2·3 to 4·1)	2·1 (1·3 to 2·9)	1·4 (0·9 to 1·8)	3·7 (2·5 to 4·9)	2·9 (1·8 to 4·0)	2·4 (1·5 to 3·2)
1930–38	1·5 (0·9 to 2·1)	0·9 (0·4 to 1·4)	0·6 (0·3 to 0·9)	2·2 (1·3 to 3·0)	1·3 (0·6 to 2·0)	0·8 (0·4 to 1·3)	2·1 (0·9 to 3·3)	1·4 (0·2 to 2·6)	1·1 (0·2 to 2·0)
1939–45	0·9 (0·3 to 1·5)	0·4 (−0·2 to 0·9)	0·2 (−0·1 to 0·6)	1·1 (0·1 to 2·1)	0·4 (−0·5 to 1·3)	0·2 (−0·4 to 0·7)	..	..	..
1946–60	−0·3 (−0·9 to 0·4)	−0·6 (−1·2 to 0·1)	−0·5 (−0·9 to −0·1)	..	..	..	..	..	..
p value for sex difference by birth cohort	0·0008	0·0032	0·0002	0·014	0·030	0·010	0·045	0·050	0·032

Data are the percentage sex difference (95% CI) in probability of functional limitations. A positive value indicates women are more likely than men to be limited. ADL=activities of daily living. IADL=instrumental activities of daily living.

* Estimates were extracted at age 65 years, 75 years, and 85 years, with age analysed as a continuous variable; analyses were further adjusted for sex, birth cohort, the interaction of sex and birth cohort, marital status, study, and region.

The increased probability of IADL and mobility limitations in women, compared to men, in all birth cohorts and ADL limitations in women in the oldest three birth cohorts was attenuated after adjustment for education and further attenuated after adjustment for labour force status (table 2). The greatest attenuation in sex differences was seen for IADL and ADL limitations, for which sex differences were approximately halved after adjustment for socioeconomic factors (eg, at age 75 years in the 1930–38 birth cohort, the sex difference in IADL limitations was 3·6%, 95% CI 2·7–4·4 before adjustment and 1·7%, 1·2–2·2 after adjustment). This attenuation was lower for sex differences in mobility limitations (eg, at age 75 years in the 1930–38 birth cohort, the sex difference was 15·1%, 13·9–16·3 before adjustment and 13·6%, 12·4–14·9 after adjustment) and no attenuation was observed at age 85 years in both the 1930–38 and 1895–1929 birth cohorts.

Adjustment for self-reported chronic conditions (appendix p 14) further attenuated sex differences in IADL and ADL limitations but only to a small extent for mobility limitations (table 3). Analyses done separately by region showed some regional variation in sex differences in functional limitations (appendix pp 8–10), particularly for ADL in Northern Europe, where men were more likely to have limitations.

**Table 3:** Role of chronic conditions in sex differences in ADL, IADL, and mobility limitations

	**At age 65 years**		**At age 75 years**		**At age 85 years**	
	Adjusted for socioeconomic factors*	Additionally adjusted for chronic conditions†	Adjusted for socioeconomic factors*	Additionally adjusted for chronic conditions†	Adjusted for socioeconomic factors*	Additionally adjusted for chronic conditions†
**≥1 mobility limitation**						
1895–1929	..	..	14·3 (12·7 to 15·9)	13·5 (12·1 to 14·9)	11·9 (10·4 to 13·3)	13·2 (11·7 to 14·6)
1930–38	11·3 (10·1 to 12·5)	9·8 (8·7 to 10·9)	13·6 (12·4 to 14·9)	11·0 (10·0 to 12·0)	11·0 (9·5 to 12·6)	11·4 (9·7 to 13·0)
1939–45	12·2 (11·1 to 13·3)	8·9 (8·0 to 9·8)	13·9 (12·4 to 15·5)	9·7 (8·4 to 10·9)	..	..
1946–60	9·3 (8·1 to 10·5)	6·1 (5·2 to 7·0)	..	..	..	..
p value for sex difference by birth cohort	0·0014	<0·0001	0·81	0·0002	0·43	0·11
**≥1 IADL limitation**						
1895–1929	..	..	1·7 (1·1 to 2·2)	1·2 (0·8 to 1·6)	4·3 (3·2 to 5·5)	3·6 (2·7 to 4·5)
1930–38	0·8 (0·5 to 1·1)	0·5 (0·3 to 0·6)	1·7 (1·2 to 2·3)	0·8 (0·5 to 1·0)	3·8 (2·4 to 5·1)	2·4 (1·4 to 3·3)
1939–45	1·0 (0·7 to 1·4)	0·3 (0·1 to 0·5)	1·8 (1·1 to 2·5)	0·5 (0·2 to 0·7)	..	..
1946–60	0·8 (0·4 to 1·3)	0·2 (0·1 to 0·4)	..	..	..	..
p value for sex difference by birth cohort	0·58	0·28	0·95	0·0067	0·48	0·023
**≥1 ADL limitation**						
1895–1929	..	..	1·4 (0·9 to 1·8)	0·8 (0·5 to 1·1)	2·4 (1·5 to 3·2)	1·4 (0·8 to 1·9)
1930–38	0·6 (0·3 to 0·9)	0·2 (0·0 to 0·4)	0·8 (0·4 to 1·3)	0·2 (0·0 to 0·4)	1·1 (0·2 to 2·0)	0·2 (−0·2 to 0·7)
1939–45	0·2 (−0·1 to 0·6)	−0·2 (−0·3 to 0·0)	0·2 (−0·4 to 0·7)	−0·2 (−0·4 to 0·0)	..	..
1946–60	−0·5 (−0·9 to −0·1)	−0·4 (−0·5 to −0·2)	..	..	..	..
p value for sex difference by birth cohort	0·0002	0·0001	0·010	<0·0001	0·032	0·0005

Data are the percentage sex difference (95% CI) in probability of functional limitations. A positive value indicates women are more likely than men to be limited. ADL=activities of daily living. IADL=instrumental activities of daily living.

* Estimates extracted at age 65 years, 75 years, and 85 years, with age analysed as a continuous variable; analyses were further adjusted for sex, birth cohort, the interaction of sex and birth cohort, marital status, study, region, education, and labour force status.

† Additionally adjusted for high blood pressure, diabetes, cancer, lung disease, psychiatric illness, arthritis, and cardiovascular disease (heart attack or stroke).

**Figure 2:**
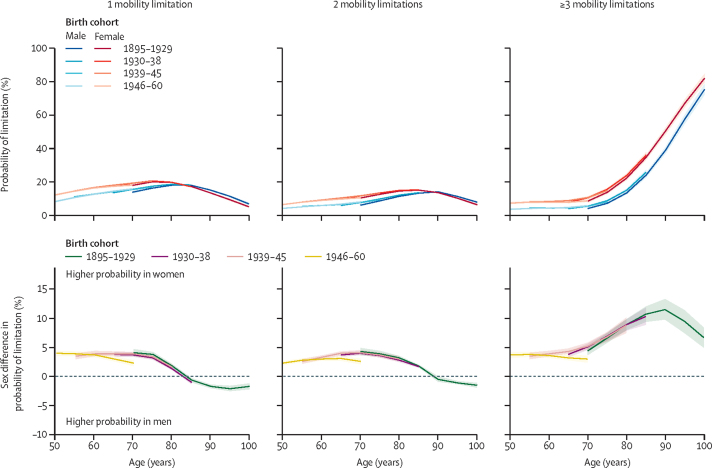
Sex differences in probability of mobility limitations by number of limitations The top panels show the probability of the given number of mobility limitations plotted by age for men and women in each birth cohort. The bottom panels show the sex difference in the probability of having a limitation. Positive values indicate women have a greater probability than men of having a given number of limitations. Predicted probabilities are based on models adjusted for sex, age, birth cohort, and their interactions, marital status, study, region, education, and labour force status and plotted for reference categories for all covariates.

Participants with mobility limitations were most likely to have one mobility limitation until age 75 years in women and age 85 years in men (figure 2). By age 80 years in women and 85 years in men, participants with mobility limitations were most likely to have three or more mobility limitations. Analyses adjusted for socioeconomic factors showed that between the ages of 50 years and 80 years, women were more likely than men to have mobility limitations, irrespective of the number of limitations (figure 2; appendix p 15). Between the ages of 70 years and 90 years, the sex difference in having three or more limitations increased markedly (at age 70 years in the 1895–1929 birth cohort the sex difference was 4·4%, 95% CI 3·7–5·2; at age 90 years it was 11·5%, 9·8–13·3) and sex differences in one and two mobility limitations decreased, such that men were more likely than women to report one limitation after age 85 years.

**Figure 3:**
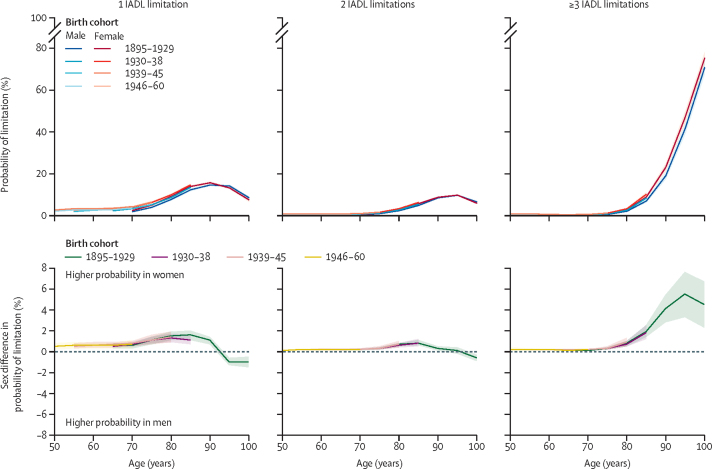
Sex differences in probability of IADL limitations by number of limitations The top panels show the probability of having the given number of IADL limitations plotted by age for men and women in each birth cohort. The bottom panels show the sex difference in the probability of having limitations. Positive values indicate women have a greater probability than men of a given number of limitations. Predicted probabilities are based on models adjusted for sex, age, birth cohort, and their interactions, marital status, study, region, education, and labour force status and plotted for reference categories for all covariates. IADL=instrumental activities of daily living.

Both men and women with IADL limitations were most likely to have one limitation until age 80 years and three or more limitations by age 90 years across birth cohorts (figure 3). IADL limitations increased from age 70 years in both men and women and sex differences in one and two IADL limitations increased progressively with age, peaking at around age 85 years (eg, in the 1895–1929 birth cohort, the sex difference at 85 years in one limitation was 1·6%, 95% CI 1·1–2·0 and in two limitations was 0·8%, 0·6–1·1; appendix p 16). Sex differences in one and two limitations decreased after age 85 years, and by age 90 years men were more likely to have one or two functional limitations, compared with women. From age 75 years, women were increasingly more likely than men to have three or more IADL limitations (eg, in the 1895–1929 birth cohort, the sex difference at age 75 years was 0·3%, 95% CI 0·2–0·4 and at age 90 years was 4·1%, 2·8–5·4).

**Figure 4:**
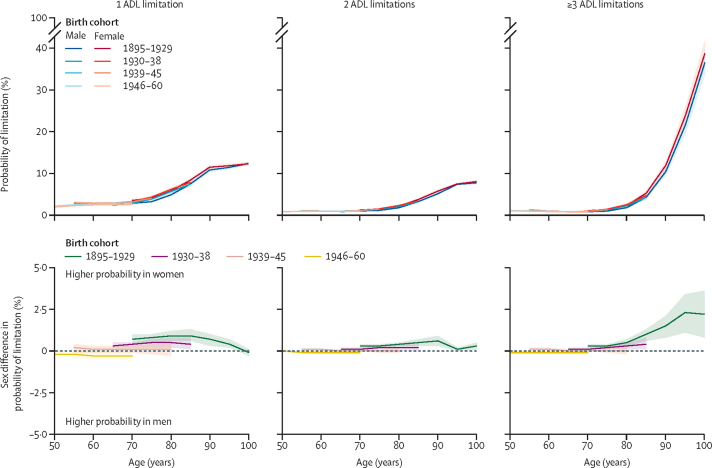
Sex differences in probability of ADL limitations by number of limitations The top panels show the probability of having the given number of ADL limitations plotted by age for men and women in each birth cohort. The bottom panels show the sex difference in the probability of having limitations. Positive values indicate women have a greater probability than men of a given number of limitations. Predicted probabilities are based on models adjusted for sex, age, birth cohort, and their interactions, marital status, study, region, education, and labour force status and plotted for reference categories for all covariates. ADL=activities of daily living.

For ADL limitations, there were no sex differences in the 1938–45 and 1946–60 birth cohorts, but women were more likely than men to have one, two, or three or more ADL limitations in the 1895–1929 birth cohort for all ages for which data were available (sex difference in three or more limitations at age 85 years was 1·0%, 95% CI 0·6–1·3) and in the 1930–38 birth cohort (0·4%, 0·1–0·8) (figure 4; appendix p 17). Sex differences in ADL limitations were stable with age, except in the oldest birth cohort.

## Discussion

In this study of 62 375 participants born between 1895 and 1960 in 14 countries with data on functional limitations spanning up to 17 years, we present three key findings. First, sex differences in ADL and IADL limitations were small across birth cohorts, particularly before age 80 years. When socioeconomic factors were considered, these differences were attenuated for IADL and eliminated for ADL in most recent birth cohorts. The sex difference in ADL was attenuated in the oldest two birth cohorts (1895–1929 and 1930–38), and eliminated in the more recent cohorts (1939–45 and 1946–60). Second, mobility limitations were higher in women across birth cohorts and ages, even after adjustment for socioeconomic factors and self-reported chronic conditions. Third, consideration of the number of limitations suggested that at the oldest ages, women were more likely to have three or more mobility and IADL limitations and men were more likely to have one or two limitations. These findings highlight the importance of considering age, birth cohort, and type and number of limitations in understanding sex differences in functional limitations.

A strength of our study is the consideration of sex differences in mobility, IADL, and ADL limitations, along with the severity of these limitations. Dichotomous categorisation (0 and 1 or more limitations) of measures of functional limitations results in loss of information by grouping together individuals with different limitation severity, as limitation in a single ADL has different implications for quality of life than does limitation in three or more ADLs. Another strength of our analysis is the use of multi-cohort data, providing a large sample size that covers a broad range of birth years and age groups. This allowed sufficient numbers in our analyses to identify trends in sex differences in functional limitations by birth cohort. A further strength is that our results reflect absolute, rather than relative, measures of sex differences, providing a more realistic measure of sex differences. The difference in probability of limitations of 1% or less, as found in many instances, might appear large when assessed using relative measures.

Our study has several limitations. First, results using self-reported measures of functional limitations might differ from results using objective measures, although sex differences have also been reported in objective measures of physical functioning, such as grip strength and walking speed.^5^ Second, the role of socioeconomic factors in sex differences in functional limitations might partly arise out of gender roles rather than biological sex; insufficient data on gender does not allow us to explore this issue further. Third, a more detailed measure of education such as number of years of schooling might better capture sex and between-country differences. Fourth, mixed effects models account for missing data for which the underlying mechanism is missing at random. It is possible that individuals with the most functional limitations were also most likely to be lost to follow-up. Nonetheless, individuals with limitations at baseline, including those in the oldest age group, were included in the analysis and the duration of follow-up was similar between men and women (mean follow-up for men was 7·2 years, SD 5·7 and for women was 7·8 years, 5·8), suggesting attrition is unlikely to have seriously affected findings for sex differences. Fifth, absence of information on dementia precludes us from considering it in the analysis, which might have led to overestimated sex differences at the oldest ages because of higher dementia rates among women.^24^ Finally, analyses by region were intended to examine variations in sex differences between regions but, given the small numbers in each region, the results should be interpreted with caution. Furthermore, a lack of information on race and ethnicity meant that we could not consider these in our analyses. Further analyses are required to examine whether the observed sex differences in functional limitations differ by race and ethnicity and by region.

In line with previous evidence,^5^ we found that women were more likely than men to report functional limitations. Part of the explanation for this finding lies in socioeconomic differences between men and women.^25^, ^26^, ^27^, ^28^ Low levels of education^29^, ^30^, ^31^, ^32^ and unemployment^29^ are associated with incident disability as they can lead to increased exposure to health risk factors^5^, and women are disproportionately more likely to have lower education and be in unpaid or domestic roles. Sex differences in chronic conditions might also play a role in sex differences in functional limitations. The attenuation of sex differences in ADL and IADL limitations after adjustment for socioeconomic factors and chronic conditions suggests that women living longer with disabilities than men^4^ is not solely responsible for observed sex differences in disability. Our finding that at older ages, when limitations were highly prevalent, women were more likely than men to have three or more IADL and mobility limitations, whereas men were more likely to have one or two limitations, is a refinement of previous findings using composite scores of limitations in ADL and IADL^33^, ^34^ and mobility activities,^34^ which reported faster decline with age in ability to do these activities in women compared with men.

Higher mobility limitations in women were not explained by socioeconomic factors or self-reported chronic conditions. More research is needed to examine whether objective measures of chronic conditions and consideration of other disability-causing conditions, such as dementia, would have attenuated sex differences. It has been proposed that remaining sex differences in mobility are due to differences in body composition, such as body-mass index and skeletal muscle index.^35^

Previous studies examining sex differences in severity of limitations in Danish centenarians born between 1895 and 1915, with sample sizes of 500 participants or less, have shown reductions in ADL limitations in women in recent birth cohorts.^11^, ^16^ Another study of 3846 participants born in 1905 and 1915 did not find any change in sex differences in ADL limitations across birth cohorts.^8^ Our study extends these findings to a broader range of birth cohorts and age groups in a larger study sample. We found no evidence of sex differences in ADL limitations in more recent birth cohorts. These findings could be due to improvements in housing and working conditions, environmental accommodations, access to assistive devices, and increased access to education, health care,^16^ and reductions in unpaid labour for women during the 20th century. Our finding of negligible variation between birth cohorts in sex differences in IADL and mobility limitations is in agreement with previous evidence.^8^, ^18^

The disablement process refers to the loss of physical or cognitive function due to chronic or acute conditions leading to difficulty doing routine tasks.^36^ This process tends to follow a hierarchical progression from mobility limitations to IADL and ADL limitations, which occur as a culmination of the disablement process at advanced age.^6^ Our finding of small sex differences for ADL and IADL, accompanied by notable sex differences in mobility limitations, in midlife suggests that mobility is an important prevention target to reduce sex differences in disability at older ages. Mobility limitations precede ADL and IADL limitations and might be milder at onset, whereas ADL and IADL limitations occur at older ages, when it might be too late to intervene. Further research is needed to identify modifiable risk factors of mobility limitations in midlife.

Sex differences in functional limitations are often examined using prevalence of at least one IADL, ADL, or mobility limitation. Our study supports the previously observed higher prevalence of functional limitations among women and shows that at older ages, when limitations are most prevalent, women are more likely to be more severely limited than are men. Our findings suggest reductions in sex differences in socioeconomic disadvantages in more recent birth cohorts are implicated in reducing sex differences in IADL and ADL. Nonetheless, efforts to reduce sex differences in disability should also focus on identifying and targeting drivers of sex disparities in mobility limitations from midlife onward, as mobility limitations might signify the beginning of a progressive disablement process.

## Data sharing

English Longitudinal Study of Ageing data are freely available to researchers through the UK data service. Irish Longitudinal Study on Ageing data are available by ISSDA application at https://www.ucd.ie/issda/data/tilda/. Survey of Health, Ageing and Retirement in Europe (SHARE) data are accessible after registration with the SHARE project at the following addresses: wave 1 (DOI:10.6103/SHARE.w1.710), wave 2 (DOI:10.6103/SHARE.w2.710), wave 4 (DOI:10.6103/SHARE.w4.710), wave 5 (DOI:10.6103/SHARE.w5.710), wave 6 (DOI:10.6103/SHARE.w6.710), and wave 7 (DOI:10.6103/SHARE.w7.711, 10.6103/SHARE.w8cabeta.001). Health and Retirement Study data are available upon registration with the University of Michigan at https://hrsdata.isr.umich.edu/data-products/public-survey-data.

## Declaration of interests

We declare no competing interests.
