# Supplementary material for: Sex differences in functional limitations and the role of socioeconomic factors: a multi-cohort analysis
Source: Lancet Healthy Longev. 2021 Dec;2(12):e780–90. doi: 10.1016/S2666-7568(21)00249-X (PMC8636280; doi:10.1016/S2666-7568(21)00249-X)
Supplement: Supplementary appendix [file mmc1.pdf]

THE LANCET
Healthy Longevity

Supplementary appendix

This appendix formed part of the original submission and has been peer reviewed. We post it as supplied by the authors.

Supplement to: Bloomberg M, Dugravot A, Landré B, et al. Sex differences in functional limitations and the role of socioeconomic factors: a multi-cohort analysis. *Lancet Healthy Longev* 2021; published online Nov 25. https://doi.org/10.1016/S2666-7568(21)00249-X.

Supplementary materials

Sex differences in functional limitations and the role of socioeconomic factors: a multi-cohort analysis

Mikaela Bloomberg, MSc[1]

Aline Dugravot, MSc[2]

Dr Benjamin Landré, PhD[2]

Professor Annie Britton, PhD[1]

Professor Andrew Steptoe, FMedSci[3]

Professor Archana Singh-Manoux, PhD[1,2]

Dr Séverine Sabia, PhD[1,2]

[1]Department of Epidemiology and Public Health, University College London, UK

[2]Université de Paris, Inserm U1153, Epidemiology of Ageing and Neurodegenerative diseases, France

[3]Department of Behavioural Science and Health, University College London, UK

Figures

Figure S1. Flowchart of sample selection in ELSA. 3
Figure S2. Flowchart of sample selection in TILDA. 4
Figure S3. Flowchart of sample selection in SHARE. 5
Figure S4. Flowchart of sample selection in HRS. 6
Figure S5. Observed proportion ≥1 mobility, IADL, and ADL limitation in ELSA, TILDA, SHARE, and HRS. 7
Figure S6. Sex differences in probability of ≥1 mobility limitation by region. 9
Figure S7. Sex differences in probability of ≥1 IADL limitation by region. 10
Figure S8. Sex differences in probability of ≥1 ADL limitation by region. 11

Tables

Table S1. Countries in each included study. 13
Table S2. Waves and years included in analyses from ELSA, TILDA, SHARE, and HRS. 14
Table S3. Activities assessed for each functional measure. 15
Table S4. Baseline distribution of self-reported chronic conditions in the pooled study population. 16
Table S5. Sex differences in probability of mobility limitations by severity of limitations. 17
Table S6. Sex differences in probability of instrumental activity of daily living (IADL) limitations by severity of limitations. 18
Table S7. Sex differences in probability of activity of daily living (ADL) limitations by severity of limitations 19

Figure S1. Flowchart of sample selection in ELSA.

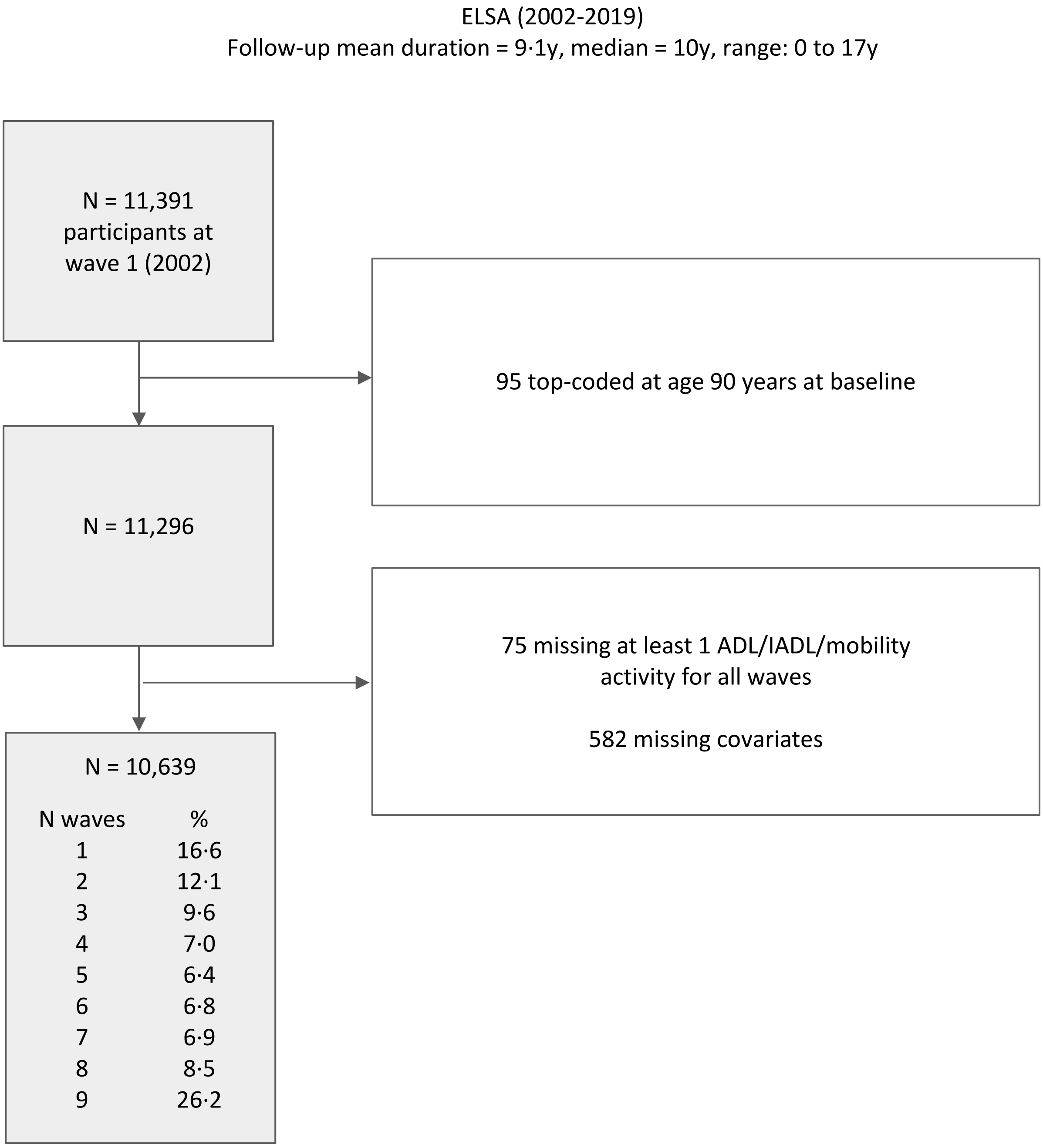

Figure S2. Flowchart of sample selection in TILDA.

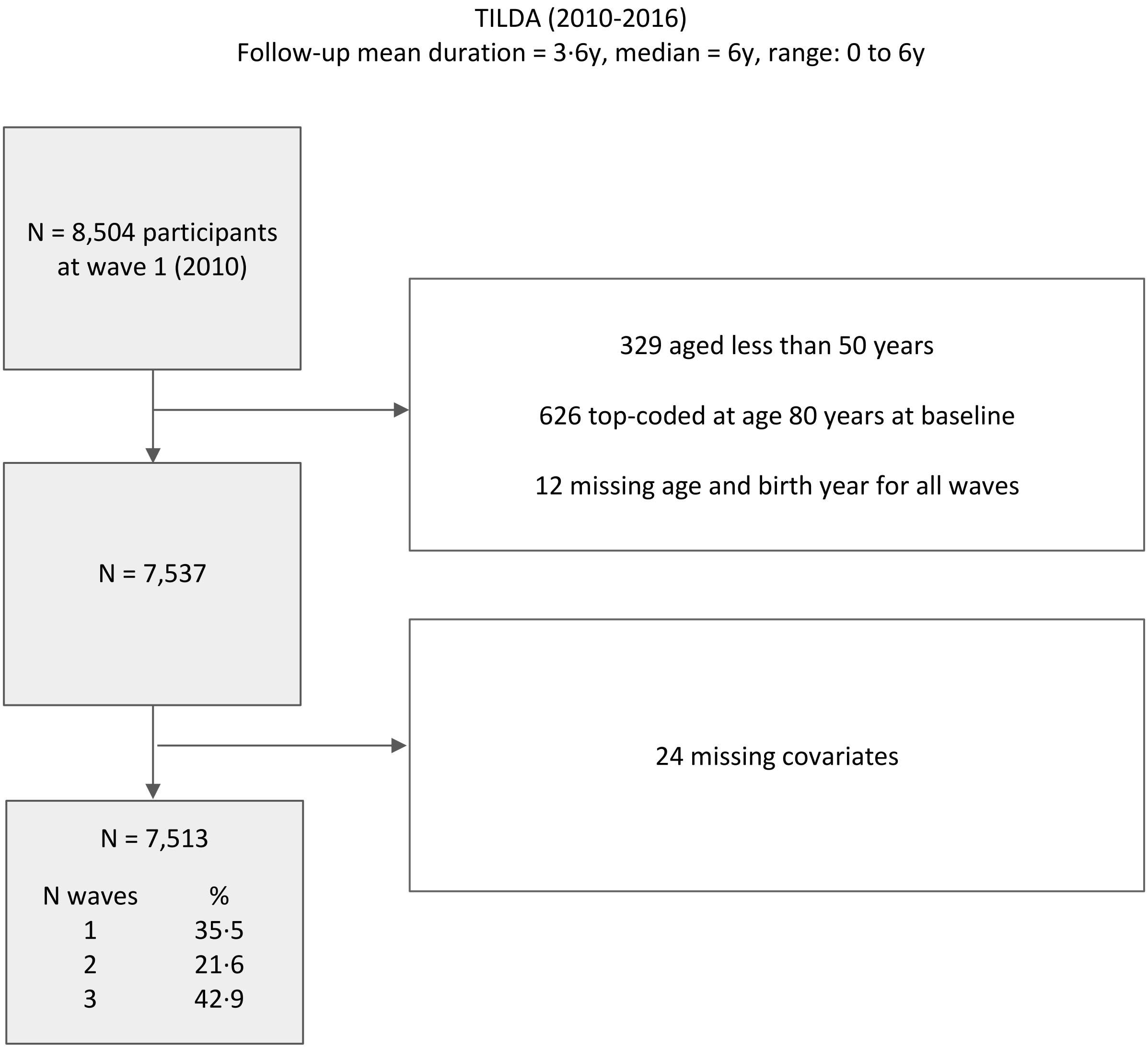

Figure S3. Flowchart of sample selection in SHARE.

SHARE (2004-2017)
Follow-up mean duration = 6·6y, median = 7y, range: 0 to 13y

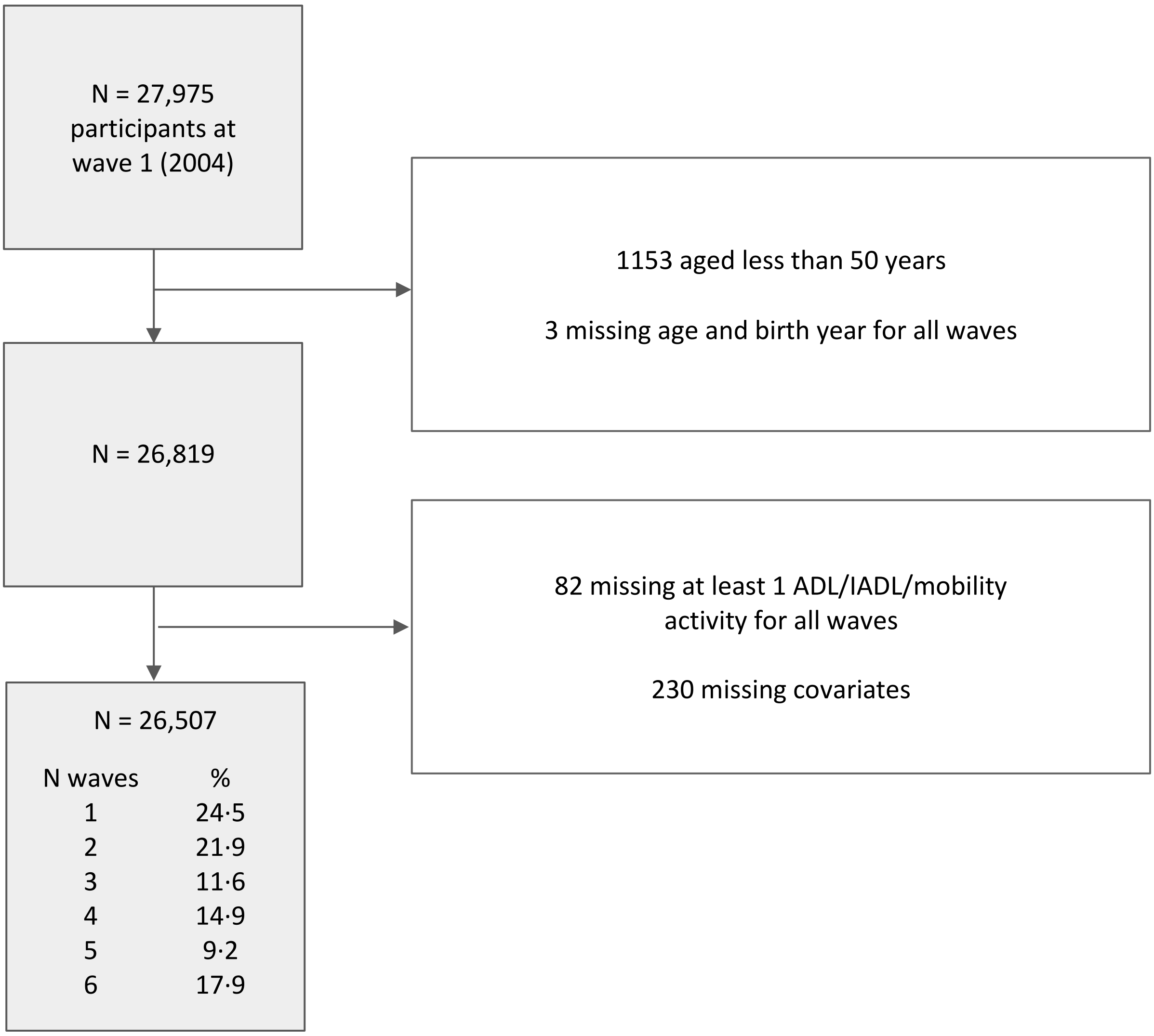

9

Figure S4. Flowchart of sample selection in HRS.

HRS (2000-2017)
Follow-up mean duration = 9·7y, median = 12y, range: 0 to 17y

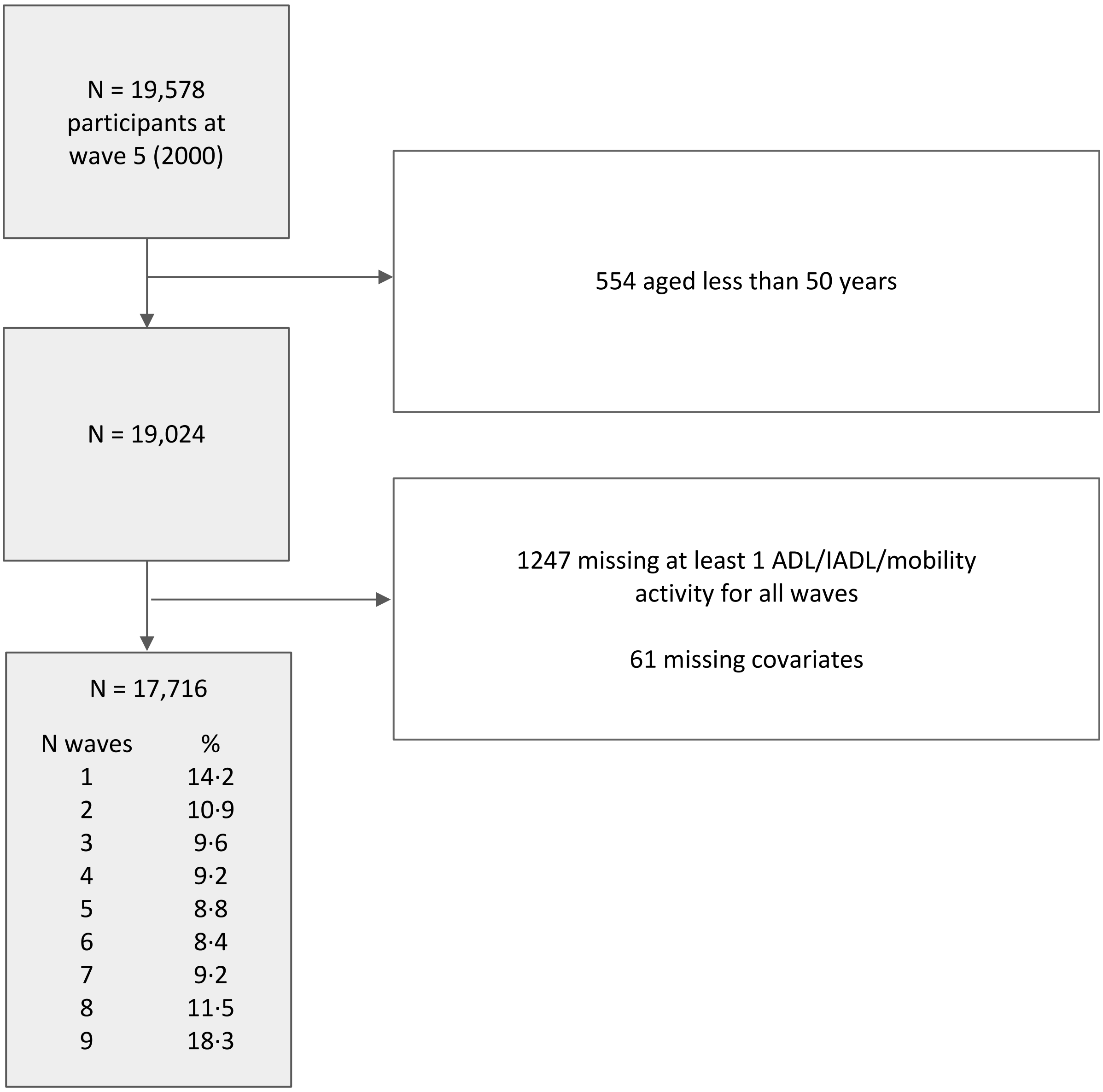

Figure S5. Observed proportion $\geq$1 mobility, IADL, and ADL limitation in ELSA, TILDA, SHARE, and HRS.

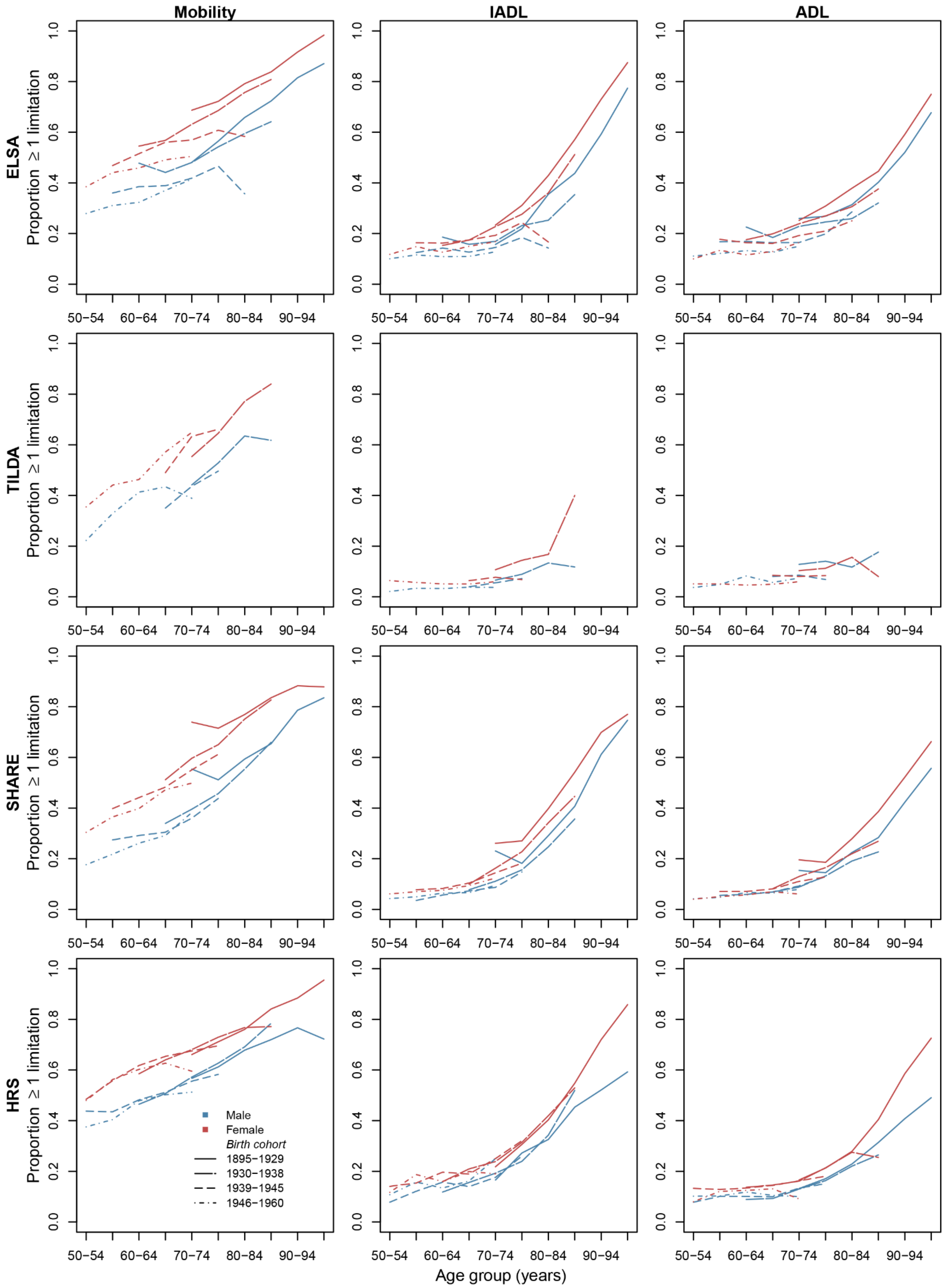
Mobility
IADL
ADL
ELSA
TILDA
SHARE
HRS
Proportion ≥ 1 limitation
0.0
0.2
0.4
0.6
0.8
1.0
50−54
60−64
70−74
80−84
90−94
Male
Female
Birth cohort
1895−1929
1930−1938
1939−1945
1946−1960
Age group (years)

Figure S6. Sex differences in probability of ≥1 mobility limitation by region.

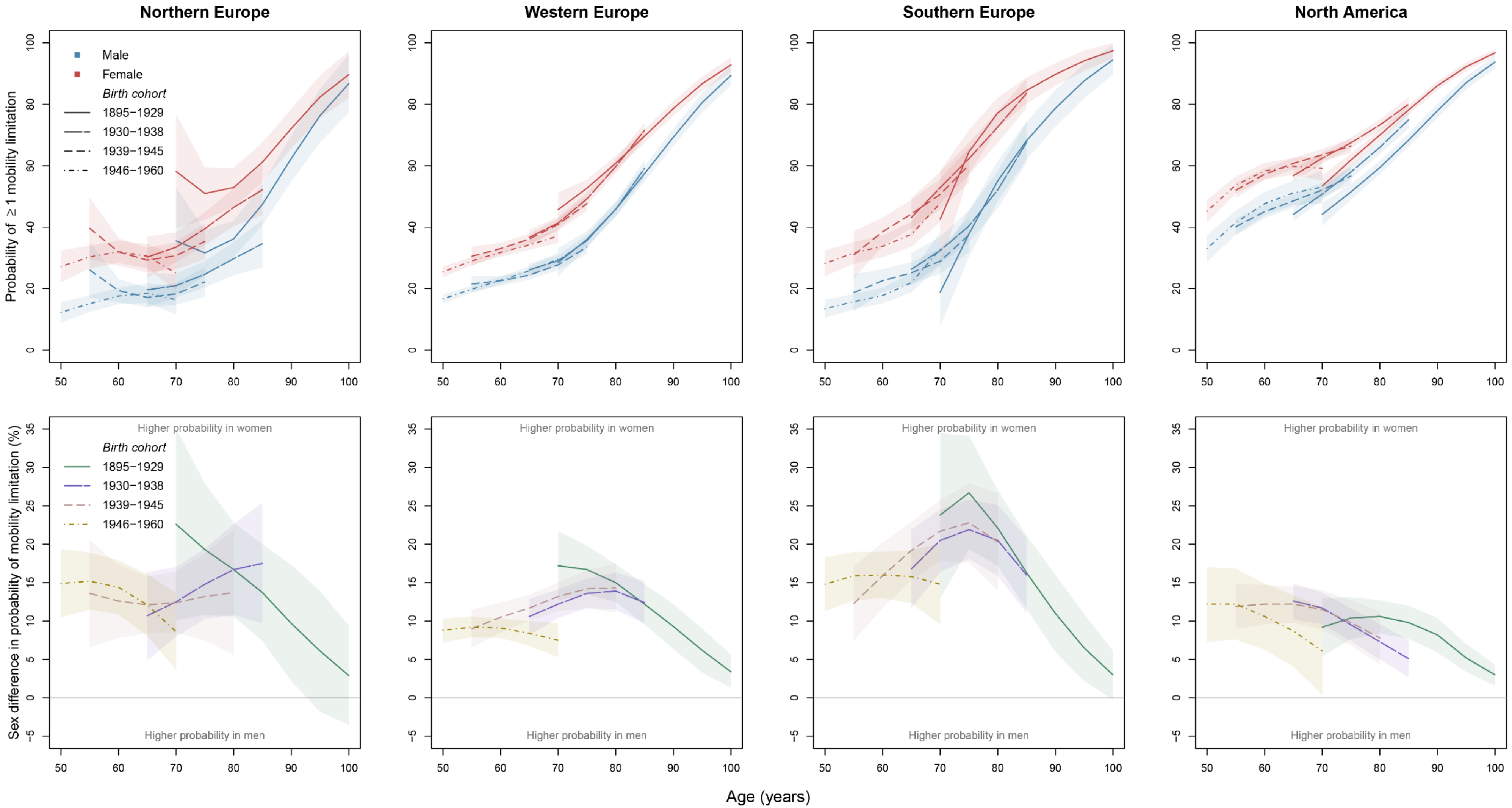

Top panel shows the probability of having ≥1 mobility limitation plotted by age for men and women in each birth cohort. Bottom panel shows the sex difference in probability of ≥1 mobility limitation: positive value indicates women have greater probability than men of ≥1 mobility limitation. Predicted probabilities based on models in each region adjusted for sex, age, birth cohort, and their interactions, marital status, education and labour force status, and plotted for reference categories for all covariates.

Figure S7. Sex differences in probability of ≥1 IADL limitation by region.

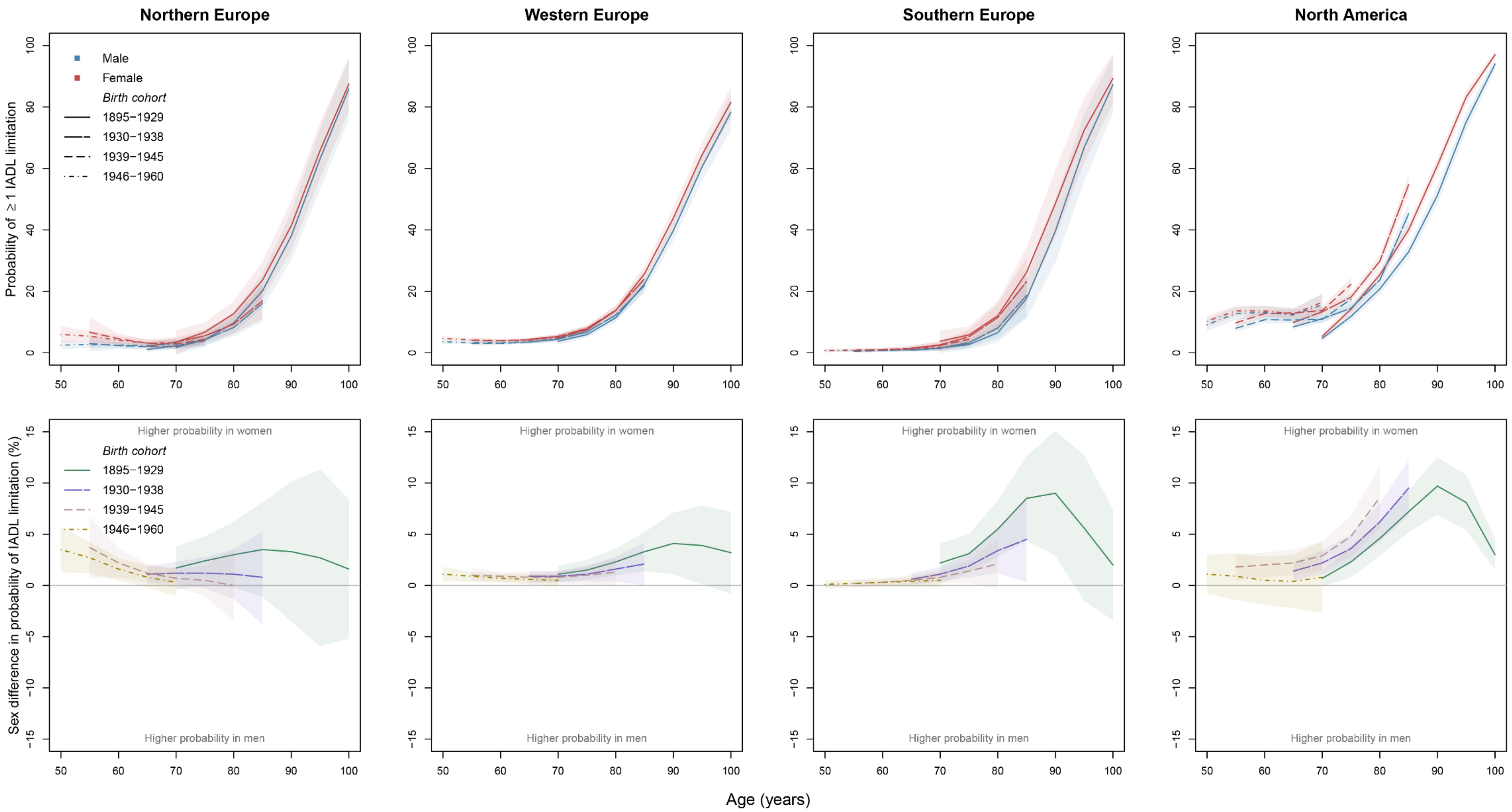

Top panel shows the probability of having ≥1 IADL limitation plotted by age for men and women in each birth cohort. Bottom panel shows the sex difference in probability of ≥1 IADL limitation: positive value indicates women have greater probability than men of ≥1 IADL limitation. Predicted probabilities based on models in each region adjusted for sex, age, birth cohort, and their interactions, marital status, education and labour force status, and plotted for reference categories for all covariates.

Figure S8. Sex differences in probability of ≥1 ADL limitation by region.

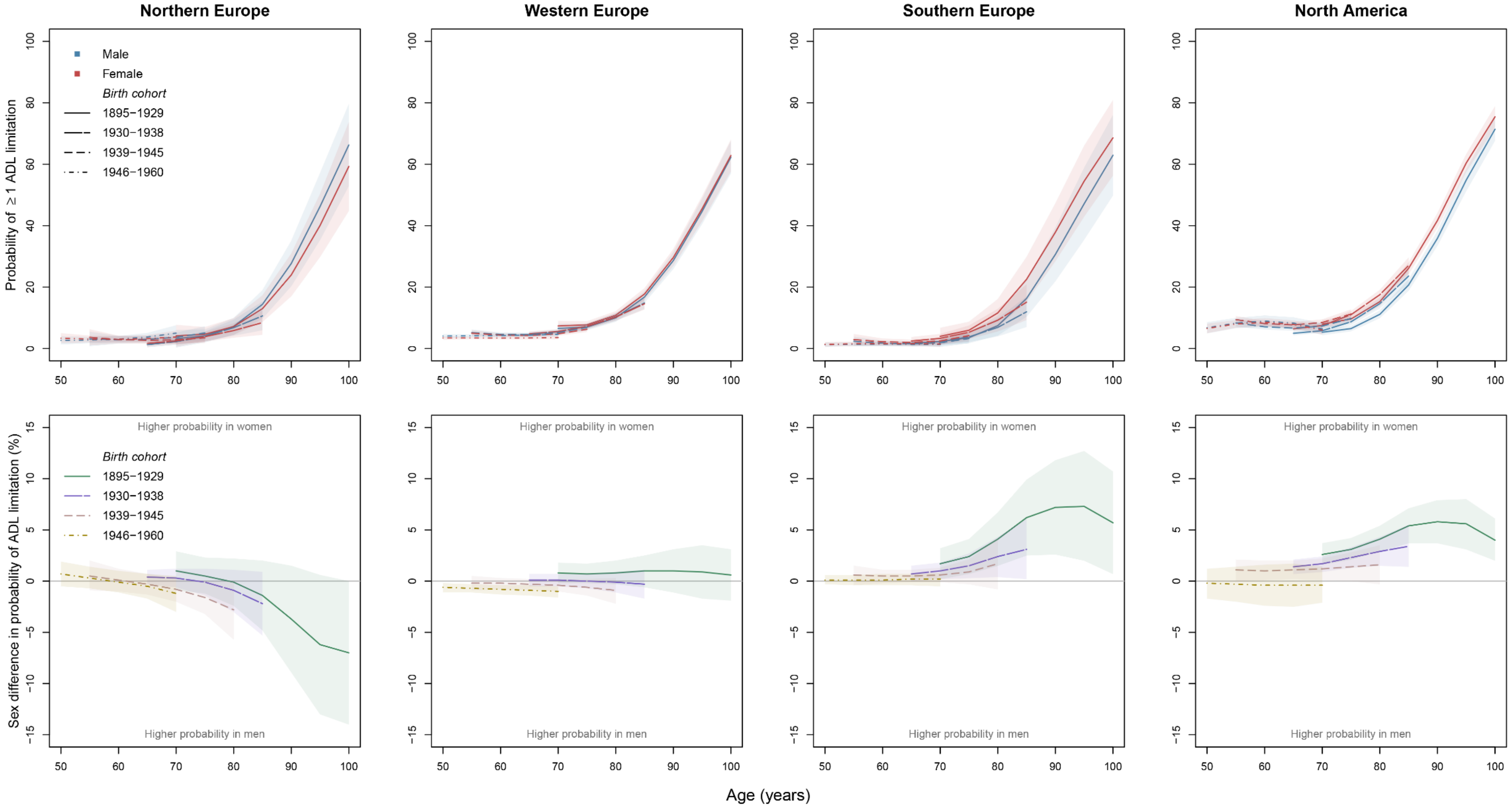

Top panel shows the probability of having ≥1 ADL limitation plotted by age for men and women in each birth cohort. Bottom panel shows the sex difference in probability of ≥1 ADL limitation: positive value indicates women have greater probability than men of ≥1 ADL limitation. Predicted probabilities based on models in each region adjusted for sex, age, birth cohort, and their interactions, marital status, education and labour force status, and plotted for reference categories for all covariates.

Table S1. Countries in each included study.

Study	*Region*	*Country*
ELSA	Western Europe	England
TILDA	Western Europe	Ireland
SHARE	Northern Europe	Denmark
		Sweden
	Western Europe	Austria
		Belgium
		France
		Germany
		Netherlands
		Switzerland
	Southern Europe	Greece
		Italy
		Spain
HRS	North America	United States

Abbreviations: ELSA: English Longitudinal Study of Ageing; TILDA: The Irish Longitudinal Study on Ageing; SHARE: Survey of Health, Ageing and Retirement in Europe; HRS: Health and Retirement Study

Table S2. Waves and years included in analyses from ELSA, TILDA, SHARE, and HRS.

Year	*ELSA*	*TILDA*	*SHARE*	*HRS*
2000				Wave 5
2001				
2002	Wave 1			Wave 6
2003				
2004	Wave 2		Wave 1	Wave 7
2005				
2006	Wave 3		Wave 2	Wave 8
2007				
2008	Wave 4			Wave 9
2009				
2010	Wave 5	Wave 1		Wave 10
2011			Wave 4	
2012	Wave 6			Wave 11
2013			Wave 5	
2014	Wave 7	Wave 3		Wave 12
2015			Wave 6	
2016	Wave 8	Wave 4		Wave 13
2017			Wave 7	
2018	Wave 9			
2019				

Abbreviations: ELSA: English Longitudinal Study of Ageing; TILDA: The Irish Longitudinal Study on Ageing; SHARE: Survey of Health, Ageing and Retirement in Europe; HRS: Health and Retirement Study

Wave 2 of TILDA and wave 3 of SHARE were included as no data on functional limitations was assessed at these waves. The baseline wave for HRS was wave 5 to allow for similar years of follow-up between studies.

Table S3. Activities assessed for each functional measure.

Mobility activities	***IADL***	***ADL***
Getting up from a chair	Managing money	Walking across the room
Climbing 1 flight of stairs	Taking medications	Dressing
Stooping, kneeling, or crouching	Grocery shopping	Bathing
Reaching/extending the arms	Preparing meals	Eating
Lifting/carrying weights over 10 lbs	Using the telephone	Getting in/out of bed
Walking 1 block/100 yds/100 m	House/garden work*	Using the toilet

*For house/garden work, HRS participants were asked whether their health limited their ability to perform housework (yes/no).

Table S4. Baseline distribution of self-reported chronic conditions in the pooled study population.

	Men	Women	P-value
	**N = 27923**	**N = 34452**	
**High blood pressure, N (%)**			
No	17795 (63·7)	21054 (61·1)	*<0·0001*
Yes	10128 (36·3)	13398 (38·9)	
**Diabetes, N (%)**			
No	24674 (88·4)	31140 (90·4)	*<0·0001*
Yes	3249 (11·6)	3312 (9·6)	
**Cancer, N (%)**			
No	26042 (93·3)	31720 (92·1)	*<0·0001*
Yes	1881 (6·7)	2732 (7·9)	
**Lung disease, N (%)**			
No	26162 (93·7)	32564 (94·5)	*<0·0001*
Yes	1761 (6·3)	1888 (5·5)	
**Psychiatric illness, N (%)**			
No	26350 (94·4)	30874 (89·6)	*<0·0001*
Yes	1573 (5·6)	3578 (10·4)	
**Arthritis, N (%)**			
No	20859 (74·7)	21504 (62·4)	*<0·0001*
Yes	7064 (25·3)	12948 (37·6)	
**Cardiovascular disease,* N (%)**			
No	21640 (77·5)	28799 (83·6)	*<0·0001*
Yes	6283 (22·5)	5653 (16·4)	

*Heart attack and stroke.

Table S5. Sex differences in probability of mobility limitations by severity of limitations.

	Percent sex difference (95% CI) in probability of number of mobility limitations		
	At age 65	At age 75	At age 85
**1 limitation**			
1895-1929	No data	3·8 (3·3, 4·2)	-0·6 (-1·0, -0·3)
1930-1938	3·8 (3·4, 4·2)	3·2 (2·9, 3·5)	-1·0 (-1·3, -0·7)
1939-1945	3·9 (3·5, 4·3)	3·4 (3·0, 3·8)	No data
1946-1960	3·0 (2·6, 3·4)	No data	No data
P sex difference by birth cohort	*0·0016*	*0·083*	*0·023*
**2 limitations**			
1895-1929	No data	3·9 (3·5, 4·4)	1·8 (1·6, 2·0)
1930-1938	3·7 (3·4, 4·1)	3·6 (3·3, 3·9)	1·7 (1·5, 1·9)
1939-1945	4·0 (3·6, 4·3)	3·7 (3·3, 4·1)	No data
1946-1960	3·1 (2·7, 3·4)	No data	No data
P sex difference by birth cohort	*0·0018*	*0·46*	*0·51*
**≥3 limitations**			
1895-1929	No data	6·6 (5·8, 7·4)	10·7 (9·4, 12·0)
1930-1938	3·8 (3·3, 4·2)	6·9 (6·2, 7·5)	10·3 (8·9, 11·8)
1939-1945	4·3 (3·9, 4·8)	6·8 (6·0, 7·6)	No data
1946-1960	3·2 (2·8, 3·7)	No data	No data
P sex difference by birth cohort	*0·00080*	*0·85*	*0·67*

Estimates extracted at age 65, 75, and 85 with age analysed as a continuous term; analyses further adjusted for sex, birth cohort, and their interactions, marital status, study, region, education, and labour force status. Positive value indicates women have greater probability than men of having given number of limitations.

Table S6. Sex differences in probability of instrumental activity of daily living (IADL) limitations by severity of limitations.

	**Percent sex difference (95% CI) in probability of number of IADL limitations**		
	**At age 65**	**At age 75**	**At age 85**
**1 limitation**			
1895-1929	No data	1·1 (0·7, 1·4)	1·6 (1·1, 2·0)
1930-1938	0·5 (0·4, 0·7)	1·1 (0·8, 1·4)	1·1 (0·7, 1·6)
1939-1945	0·7 (0·4, 0·9)	1·1 (0·7, 1·6)	No data
1946-1960	0·6 (0·3, 0·8)	No data	No data
P sex difference by birth cohort	*0·63*	*0·97*	*0·098*
**2 limitations**			
1895-1929	No data	0·3 (0·2, 0·4)	0·8 (0·6, 1·1)
1930-1938	0·2 (0·1, 0·2)	0·3 (0·2, 0·4)	0·8 (0·5, 1·2)
1939-1945	0·2 (0·1, 0·3)	0·3 (0·2, 0·5)	No data
1946-1960	0·2 (0·1, 0·2)	No data	No data
P sex difference by birth cohort	*0·57*	*0·97*	*0·99*
**≥3 limitations**			
1895-1929	No data	0·3 (0·2, 0·4)	1·9 (1·4, 2·5)
1930-1938	0·1 (0·1, 0·2)	0·3 (0·2, 0·4)	1·8 (1·2, 2·5)
1939-1945	0·2 (0·1, 0·2)	0·3 (0·2, 0·5)	No data
1946-1960	0·1 (0·1, 0·2)	No data	No data
P sex difference by birth cohort	*0·39*	*0·78*	*0·74*

Estimates extracted at age 65, 75, and 85 with age analysed as a continuous term; analyses further adjusted for sex, birth cohort, and their interactions, marital status, study, region, education, and labour force status. Positive value indicates women have greater probability than men of having given number of limitations.

Table S7. Sex differences in probability of activity of daily living (ADL) limitations by severity of limitations.

	Percent sex difference (95% CI) in probability of number of ADL limitations		
	**At age 65**	**At age 75**	**At age 85**
**1 limitation**			
1895-1929	No data	0·8 (0·5, 1·0)	0·9 (0·6, 1·3)
1930-1938	0·3 (0·2, 0·5)	0·5 (0·2, 0·7)	0·4 (0·1, 0·8)
1939-1945	0·1 (-0·1, 0·3)	0·1 (-0·2, 0·4)	No data
1946-1960	-0·3 (-0·5, -0·1)	No data	No data
P sex difference by birth cohort	*0·00020*	*0·010*	*0·035*
**2 limitations**			
1895-1929	No data	0·3 (0·2, 0·4)	0·5 (0·3, 0·7)
1930-1938	0·1 (0·1, 0·2)	0·2 (0·1, 0·3)	0·2 (0·0, 0·4)
1939-1945	0·0 (-0·0, 0·1)	0·0 (-0·1, 0·1)	No data
1946-1960	-0·1 (-0·2, -0·0)	No data	No data
P sex difference by birth cohort	*0·00020*	*0·0076*	*0·037*
**≥3 limitations**			
1895-1929	No data	0·3 (0·2, 0·4)	1·0 (0·6, 1·3)
1930-1938	0·1 (0·1, 0·2)	0·2 (0·1, 0·3)	0·4 (0·1, 0·8)
1939-1945	0·0 (-0·0, 0·1)	0·0 (-0·1, 0·2)	No data
1946-1960	-0·1 (-0·2, -0·0)	No data	No data
P sex difference by birth cohort	*0·00020*	*0·012*	*0·028*

Estimates extracted at age 65, 75, and 85 with age analysed as a continuous term; analyses further adjusted for sex, birth cohort, and their interactions, marital status, study, region, education, and labour force status. Positive value indicates women have greater probability than men of having given number of limitations.
